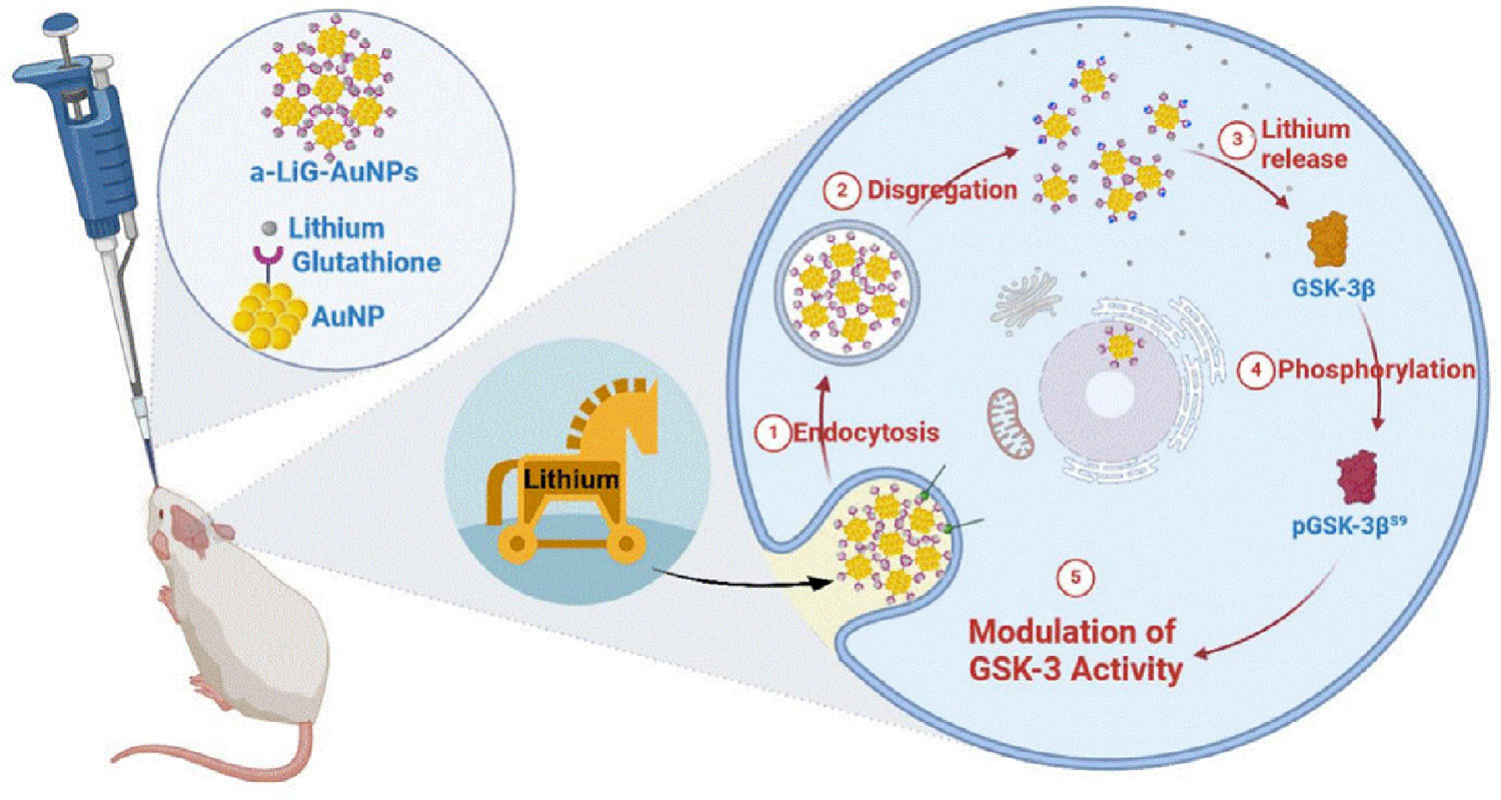# Lithiated gold nanoparticles as promising therapy for Alzheimer's disease

**DOI:** 10.1002/alz70859_097598

**Published:** 2025-12-25

**Authors:** Gulia Puliatti, Beatrice Cannata, Laura Sposito, Martina Albini, Alfonso Grassi, Antonio Buonerba, Claudio Grassi, Roberto Piacentini

**Affiliations:** ^1^ Università Cattolica del Sacro Cuore, Rome Italy; ^2^ Fondazione Policlinico Universitario A. Gemelli IRCCS, Rome Italy; ^3^ University of Salerno, Salerno Italy; ^4^ Università Cattolica del Sacro Cuore, Rome, Rome Italy

## Abstract

**Background:**

Glycogen Synthase Kinase 3β (GSK‐3β), is a hub kinase whose activity deregulation is involved in mood disorders and neurodegenerative diseases, including Alzheimer's disease (AD). Many efforts have been made to develop drugs able to modulate this kinase, but most of them did not reach the human use. The lithium cation (Li+) is a potent GSK‐3 inhibitor, that modulates kinase activity by inducing its phosphorylation at Ser9 (pGSK‐3β‐S9). Unfortunately, Li+ faces challenges in systemic use due to its toxicity, especially for kidneys and thyroid. Nowadays, lithium salts, taken orally, are used for treating neuropsychiatric disorders, but safe doses are ineffective against other GSK‐3‐dependent illnesses, which require higher, toxic doses. Thereby, identifying novel lithium administration routes reaching the target organ(s) (e.g., the brain) bypassing the systemic administration and without damaging other organs, would be desirable.

**Method:**

We developed a tool consisting of gold nanoparticles (AuNPs) functionalized with glutathione and charged with Li+ in the outer corona, forming aggregates in water of 250 nm in diameter (a‐LiG‐AuNPs). a‐LiG‐AuNPs internalize cells and determine an efficient intracellular release of Li+. The effects of a‐LiG‐AuNPs in modulating GSK‐3β and contrasting AD neuropathological signs have been tested on cultured cells and in mice.

**Result:**

Addition of a‐LiG‐AuNPs to the culture medium of SH‐SY5Y cells induced a significant increase of pGSK‐3β‐S9 respect to classical salt formulations, even at low Li+ concentration (0.15 mM) normally resulting ineffective with lithium salts.

If administered intranasally in healthy mice (C57BL/6; 5 days a week, twice a month, up to 5 months), a‐LiG‐AuNPs directly reached the brain and increased pGSK‐3β‐S9 in situ (+130%; p<0.01 vs. vehicle), without significantly affecting plasma Li+ levels (0.08±0.01 and 0.14±0.02 mmol/L, respectively for vehicle‐ and a‐LiG‐AuNP‐treated mice), nor inducing gliosis or affecting the general health status of the animals.

More interesting, a‐LiG‐AuNP treatment spared murine hippocampal neurons from the synaptotoxic action of tau oligomers in vitro, and significantly ameliorated the memory of 13 months‐old 3×Tg‐AD mice, evaluated by Y‐maze and Novel object recognition paradigms.

**Conclusion:**

Collectively, a‐LiG‐AuNPs offer a promising method for brain‐specific Li+ delivery, potentially beneficial for treating GSK‐3β‐dependent neurological diseases such as AD, while avoiding the Li+ side effects.